# Notes and Miscellany

**Published:** 1885-02

**Authors:** 


### NOTES AND MISCELLANY

=M. Gerard Lague (*Lyon Med.*) states that syrup of gooseberry will completely disguise the taste of iodide of potash.

=To Relieve Cramps.—Grasp the affected portion, as the calf of the leg, and press it firmly. Persons subject to this unpleasant affection should carry with them a strap which may be buckled tightly about the cramping muscle.

=Lumbago—The *Scientific American* says: Lumbago may be quickly relieved by binding a piece of enameled cloth, such as is used to cover tables, over the loins outside of the flannel shirt. Profuse perspiration is produced which rapidly relieves the pain.

=Forcing the Intellect of Children.—The *Medical and Surgical Reporter* in a recent able editorial on this subject, contends that such injury is done by sending children to school too early, and holds, very properly, we think, that the portion of life prior to puberty should be devoted mainly to physical development.

=Mr. Henderson says: "I find that turf can be successfully laid down, if necessary, in dry and hot summer weather, by simply covering it, when finished, before it gets too dry, with about a quarter of an inch of light soil put through a half inch sieve. The grass begins to grow through the soil in a very few days."

=Chloral in Chorea.—Dr. Mosler relates a case of very severe general chorea in a girl eighteen years old, in which a speedy cure was obtained by chloral. After morphine and arsenic had been given for two weeks without effect, chloral was exhibited in thirty-grain doses, at first four times, then twice, and finally once a day. On the very first day the patient slept soundly, and at the end of a week the choreic movements had ceased entirely. It is worthy of note that the onset of the chorea was preceded by the toothache, a circumstance which has been remarked by other observers.

=A Fable; The Fly and the Seals.—One Day a Fly sat on a Tree near the Pond Intently Gazing at the Playful Antics of the Seals. All at Once He said to Himself:

"I'd like to know if that Funny Business on their Sides is Fins or Feet."

So saying, he flew to see, and Perched on the Wet Breast of a Seal and stuck there, and before he could Extricate Himself, the Seal again Leaped into the Water and the Fly was Drowned.

Moral.—Don't be too Fly about Getting Stuck on People till you are Pretty Well Acquainted with their Private Habits.—*Puck.*

=CAULOCOREA.—Dr. J. J. Caldwell says in *The Brief:* I have found this a very valuable remedy in the debilities common to the female, as a nervine, tonic and alterative. The following will illustrate its remedial applications: Mrs. L., aged 46, who has been losing flesh for years past, had suffered loss of appetite, sleeplessness and nervous debility, with indigestion and at times great laxativeness of the bowels, and had tried various remedies with little or no success. At last she was recommended the use of Caulocorea, with good results. Now she has better flesh, improved appetite, and more cheerful mind, and is sc much pleased with this remedy that she desires to continue it. Many other cases might be cited to sustain the reputation of this valuable remedy, if it were necessary, as it is frequently and favorably alluded to by medical writers.

=RUST STAINS ON WASHED LINEN.—Rust stains on washed linen may be caused by a variety of circumstances, but generally the cause is to be looked for in the use of the "soluble blueing" so much used to whiten clothes. These soluble blues are, in almost every instance, composed of a chemical compound containing iron and known as "Prussian blue." This substance is decomposed by the fixed alkalies, soda (or potash), and if the clothes are not thoroughly rinsed to free them from soap suds or washing soda (if this has been used), the alkali left in the clothes will decompose the blueing and cause rusty stains to appear wherever the two may happen to adhere on the clothes in contact with each other; or it may cause the linen to take on a yellowish tint, which is often so aggravating to the painstaking housewife. To test the correctness of this explanation, we offer the suggestion, in case it should prove that soluble blue has been used in washing the clothes, that it be discarded, and the old-fashioned indigo bag used in its place.

=The Holy Well of Mecca affords Prof. Franklin the opportunity of setting forth in the London *Times*, in a very convincing manner, the way in which superstition aids in the spread of that frightful scourge of humanity, called "Asiatic cholera." This well is regarded as sacred by the followers of Mohammed, and the manner in which it serves the purpose of spreading disease is thus set forth in the article referred to: "The well is in Mecca; the water is regarded as holy, and large quantities are annually sent as gifts to all Mussulman countries. Most of the Mohammedan princes, especially those of India, have 'keepers of the well,' whose duty it is to send them annually water from it. I have analyzed this water, and find it to be of the most abominable character. In fact, it is sewage more than seven times as concentrated as London sewage, and it contains no less than 579

grains of solid matter per gallon. Knowing the composition of this water, and the mode of propagation of Asiatic cholera by excrementitious matters, it is not to be wondered at that outbreaks of this disease should often occur among pilgrims to Mecca, while it would scarcely be possible to provide a more effective means for the distribution of cholera poison throughout Mohammedan countries."

=Professor Da Costa gives the following general rules for the treatment of fevers:—

1. Reduce the temperature. The cold bath will do this most rapidly and certainly, but it is troublesome, and not altogether free from danger, and should, therefore, only be used as a last resort. Quinine in full doses is safer, and may usually be relied upon. It should not, however, be repeated too often, as it may produce alarming cerebral symptoms, with diarrhœa and general perturbation.

2. Lessen the rapidity of the circulation. Aconite is the best remedy here, especially if the pulse is full and frequent, but if the circulation is weak, digitalis will act better. Professor Da Costa does not, however, often give either. He prefers to endeavor to reduce the temperature, and so indirectly to control the circulation.

3. Keep up the secretions. Remove the waste of the tissues by diuretics, diaphoretics, and laxatives.

4. Nourish the patient. "Don't starve a fever." Give milk, beef-juice, and other light nutritious food in small quantities, but at frequent intervals. give the patient plenty of fluids also. Slightly acidulatid drinks will be found to be both grateful and beneficial.—*Medical Bulletin.*

=Never stand at the foot of a sick bed and survey the patient. All figures loom large to fevered eyes, and by the side of the bed are only partially seen, and do not annoy with the sense of too much presence. Do not open the door very slowly, for then the attention is strained, speculating as to who the next comer can possibly be after all this preparation and with such cautious approach. Low but clear tones, quiet but sure movements, and rapid, rather than slow, are a great relief to any patient who is blessed with a practical nurse. Whispering is torture. Silence is best until you can discuss matters in another room; but if you speak, speak out, and make no mysteries about anything. In severe illness the nurse must watch her patient steadily, but not seem to be looking. In convalescence it frequently soothes the invalid to have the nurse seated at the window, apparently looking out. This frees the faculties from the tension that the sense of being watched usually gives.

